# Manga reading on paper vs. digital devices: Prospective effects on core and supportive integration processes in the brain

**DOI:** 10.1371/journal.pone.0349778

**Published:** 2026-06-03

**Authors:** Keita Umejima, Yuki Sunada, Kuniyoshi L. Sakai

**Affiliations:** Department of Basic Science, Graduate School of Arts and Sciences, The University of Tokyo, Tokyo, Japan; Max Planck Institute for Empirical Aesthetics: Max Planck Institut fur empirische Asthetik, GERMANY

## Abstract

Reading on paper reportedly improves story comprehension compared to its digital version, although the underlying neuroscientific mechanisms remain unclear. We used a format of Japanese manga stories as visual narratives told in halves, each of which depicted the same events from the different perspectives of two protagonists. We examined how the medium used to read the *first halves*, either on a paper book (Paper preparatory condition) or on an electronic tablet (Tablet preparatory condition), facilitated reading of the *second halves* for the memorized stories, which they read on an electronic display with continuous empathy ratings. Magnetic resonance scanning was conducted during the latter reading and during answering two sets of questions: Set 1 that could be answered after reading the first half alone, and Set 2 that required comprehension from both halves. Behavioral results showed prospective effects of reading manga stories on a paper book or an electronic tablet, such that the response times were prolonged in Set 2 for the Tablet condition. By comparing the results of Sets 1 and 2 with correct answers for each story, we found significant response time differences for the Tablet condition alone. Moreover, for the Paper condition, activations in the *left* frontal regions significantly decreased while reading the second halves, and those in the *right* frontal regions also decreased in Set 1. Furthermore, core left frontal activations were highest in Set 2 for the Tablet condition, while supportive right frontal activations correlated with individual accuracy rates in Set 2 for the Tablet condition, indicating that excessive integration processes support improved performances required by correct answers. The present results demonstrate stronger prospective effects of reading on paper books, such that linguistic and narrative-structural integration processes are facilitated and led to saved excessive activations.

## Introduction

Reading books involves a range of complex receptive and generative processes, including comprehension and associated cognitive factors. It is also possible that the reading medium, whether a paper book or digital version (on computer/mobile devices), may affect comprehension processes. A behavioral study of university students showed that reading on paper led to higher comprehension compared with reading on an electronic display, and that the latter condition was accompanied by higher levels of stress and tiredness [1]. Another study replicated the former finding, further suggesting the multimodal information provided by paper, with *spatiotemporally* fixed cues to the text positions [2]. Reading on paper may also lead to better awareness for own task performances due to less engagement in unrelated information [3]. These factors would affect comprehension, which consists of direct information from the text, indirect information related to the text, and generated inferences [4], further affecting construction and *linguistic integration* of necessary information [5]. Neuroscientific experiments are expected to provide direct evidence for such cognitive and metacognitive processes.

In our previous functional magnetic resonance imaging (fMRI) study, we compared three groups of participants who wrote down upcoming appointments on a calendar using a paper notebook, an electronic tablet, or a smartphone [6]. We tested the participants’ recognition memory of those appointments, and the accuracy of the easier (i.e., more straightforward) questions was higher for the paper notebook group than the electronic tablet group, even though both groups handwrote on their calendars using pens. Brain activations during retrieval were stronger when the information had been *encoded* on paper notebooks compared to the electronic devices (tablet and smartphone), especially in the left language areas of the lateral premotor cortex/inferior frontal gyrus (LPMC/IFG), angular/supramarginal gyri (AG/SMG), and their right homologues, as well as in the bilateral posterior hippocampus. The differences likely arose from spatial and structural information in the calendar, which were especially abundant when participants used paper notebooks.

The benefits of paper media are not limited to the comprehension of text information itself; pictorial information such as drawings or illustrations may also be influenced by spatial and structural information during comprehension processes. In two of our previous fMRI studies, we examined the processing of *visual narratives* from Japanese manga, which provide rich pictorial information (though usually presented in monochrome) that facilitates the comprehension of scenes. In the first study, we used “silent manga,” i.e., manga drawn without text, and compared a double-page presentation (typical of manga paper books) with a single-page presentation (common on smartphones) [7]. While reading manga on a double-page spread, which conformed to original intentions of structuring individual frames, participants’ bilateral visual cortex and cerebellum activations were clearly stronger, compared to those during a single-page presentation that impaired such structures. In the second study, we measured cortical responses in the bilateral fusiform gyrus, the region associated with color processing, and found that responses to monochrome manga were comparable to those elicited by colored bars, indicating mental imagery of colors generated in a contextual flow [8].

As regards structural properties of Japanese manga works, there was a proposal stating that “sequential drawings ordered by a rule system — a grammar — literally comprise a *visual language*” [9], indicating “a *narrative grammar*” with tree structures [10]. While comprehension in natural language and visual narratives shares similar syntax-related aspects, the other cognitive process involved in manga reading would be the construction and retrieval of “story schema,” beyond such integration. Indeed, manga reading requires panel ordering, spatial relationship processing, temporal sequencing, and narrative inferencing, which closely correspond to story schema formation.

In our recent fMRI study to elucidate thinking processes, we used illustrative quizzes including completion of a three-frame story, just like manga, and found that the bilateral posterior temporal gyri were activated selective for the linguistic factor of *clausal* [11]. Another factor of thought relevant to language structure and use is *propositional*, which actually activated the L. IFG. These two factors correspond to duality of semantics [12]. Moreover, we also identified the *recursive* system including the L. LPMC/dorsal IFG and R. LPMC, which complete three syntax-related systems for both language and thought processes.

**Fig 1:**
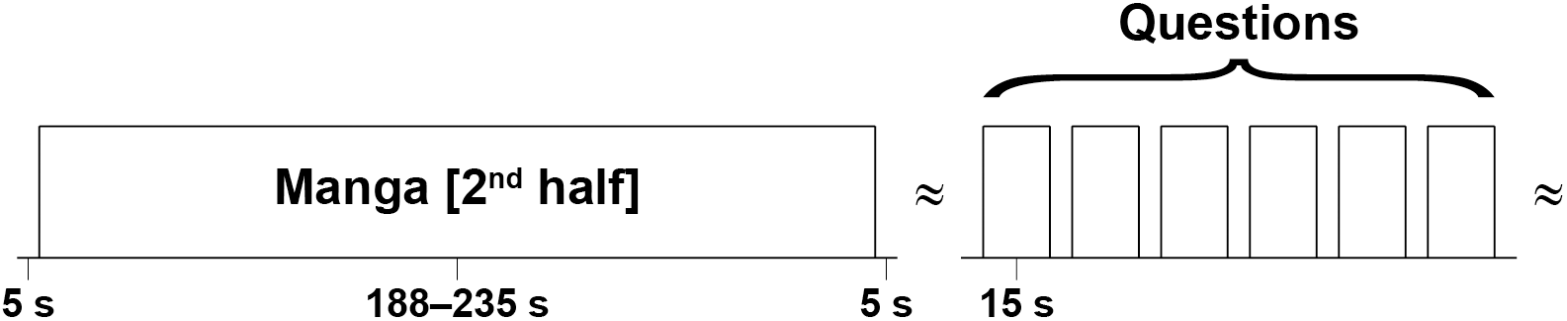
Experimental design. After the preparatory conditions of reading the *first halves* of two manga stories (either before or after the break; see Table 1), the participants received two scanning runs with the *second halves* of those manga stories (18–22 pages each; Manga condition), immediately followed by two more runs with questions on those stories (six each; Questions condition). The symbol “≈” denotes repetition.

To clarify differences between paper and electronic media for manga reading, here we compared reading manga on a paper book or electronic tablet outside an MR scanner, and assessed how this *preparatory reading* influenced subsequent manga reading, which would reveal prospective effects. We targeted the latter processes, and measured associated brain activations inside a scanner. For this purpose, we adopted a popular work of Japanese manga, in which each story consisted of a first and a second half, *repeating* the same happenings, but from the different perspectives of two protagonists. This format is known as a “zapping story,” realistically depicting conflicting feelings between the couple while preserving the same situations. For each story, we assigned the *first half* to one of the preparatory conditions (i.e., Paper or Tablet) before MR scanning, and the *second half* to the Manga condition during scanning (Table 1 and Fig 1). To monitor each participant’s active involvement in reading the stories, the Manga condition was tested with an empathy rating by the participants toward the protagonists. Because “spatial and structural information” (see above) is also useful in manga reading, we predict that comprehension and memory encoding of the stories in the first halves would be enhanced under the Paper preparatory condition. Such advantages should facilitate reading the second halves of the stories while retrieving information of each first half, and thus alleviate linguistic and narrative-structural integration under the Manga condition.

**Table 1:** The flow of story presentation.

Story	First half (outside MR scanner)	Second half (inside MR scanner)
1	I) Perspective from Ritsuko	III) Perspective from Nonchan
2	II) Perspective from Nonchan	IV) Perspective from Ritsuko
Break		
3	V) Perspective from Ritsuko	VII) Perspective from Nonchan
4	VI) Perspective from Ritsuko	VIII) Perspective from Nonchan

A popular work of manga was presented, where the first half of each story (1–4) was told from the perspective of one of two protagonists (Ritsuko and Nonchan), and the second half from the perspective of the other on the same stories. Among the first halves of the four stories, one half of the participants read stories 1 and 4 on a *paper* book (Paper preparatory condition), whereas they read stories 2 and 3 on an electronic *tablet* (Tablet preparatory condition). For the other half of the participants, we switched conditions to counterbalance between the story sets. The Roman numerals denote the order of presentation. The participants read the first halves of stories 1 and 2 outside the MR scanner (I and II) and then read the second halves of stories 1 and 2 inside the scanner (III and IV). After a 10-minute break, the procedure was repeated for stories 3 and 4 (V–VIII).The second halves were always read on an MRI-compatible electronic display (Manga condition).

Following the Manga condition, we administered questions about the stories that had been presented (Questions condition); those questions required direct/indirect information and inferences (see above). The questions were grouped into two sets: *Set 1* included questions that could be answered after reading the first halves alone, while *Set 2* included questions that required comprehension of both halves. Because Set 2 questions required referring to and integrating both halves, they would impose higher processing demands of linguistic and narrative-structural integration than Set 1. During the question phase, differences in prior reading modality (Paper vs. Tablet) would differentially influence how participants engage with Set 1 and Set 2 questions.

Previous research has clarified distinct roles for the left *core* language regions and their *supportive* right homologues in linguistic processing. In our fMRI study on grammar acquisition in a new language (Kazakh), multilingual participants showed primarily left-lateralized activation, including the L. LPMC/IFG and L. AG/SMG, when beginning to acquire the grammatical structures of simple sentences [13]. In contrast, the acquisition of complex sentences by *multilingual* participants, as well as that of both simple and complex sentences by *bilingual* participants, elicited bilateral activations, including increased engagement of the R. LPMC/IFG and R. AG/SMG, i.e., regions that support syntactic processes. These language-related regions in both hemispheres would be involved in the present study as well, corresponding to linguistic processes imposed under more demanding conditions.

## Materials and methods

### Participants

We recruited 29 undergraduate or graduate students who were native speakers of Japanese. Laterality quotients (LQ) were evaluated by the Edinburgh inventory [14]. One participant was excluded from analyses due to left-handedness (i.e., negative LQ). We also excluded three participants with either a clinical issue, orthodontic archwires, or excessive head movements in multiple runs (four out of eight runs). Among the remaining 25 participants (11 females; age: 23.5 ± 4.6 years; LQ: 89 ± 14 [mean ± SD]), 13 regularly read manga (2 hours a week on average [range: 0.4–7.0 hr]), including 8 participants who read both paper and electronic books, 2 who read the former alone, and 3 who read the latter alone.

All participants provided their written informed consent to participate in this study after the nature and possible consequences of the study were explained. These experiments (recruitment period for the participants: December 26, 2023 − September 30, 2024) were reviewed and approved by the Ethical Review Committee for Experimental Research Involving Human Subjects at the Graduate School of Arts and Sciences, The University of Tokyo, Komaba Campus (approval no. 622−8). All research procedures were performed in accordance with the Declaration of Helsinki, the Singapore Statement on Research Integrity, and the relevant guidelines/regulations in Japan (the Science Council of Japan and the Japan Society for the Promotion of Science).

### Stimuli

We used the first volume of “Kuuneru Futari, Sumu Futari (The two eating, sleeping, and living together),” a manga by Kinoko Higurashi, published by COAMIX. We selected stories 1, 2, 3, and 4, whose first halves were 20, 20, 21, and 18 pages long, respectively, whereas their second halves were 20, 22, 17, and 20 pages long, respectively. The manga was always read on a double-page spread, except for the single-page presentation of the last pages for story 3 (both first and second halves).

For the first halves, the participants read stories on either a paper book [size 24.4 × 18.2 cm² when opened] or on an electronic tablet [Microsoft Surface Pro X 13 inch (2019), presentation size 25.5 × 18.2 cm² in landscape orientation]. Among the 4 stories, half of the participants read stories 1 and 4 on a paper book (Paper preparatory condition) and stories 2 and 3 on a tablet (Tablet preparatory condition). For the other half of the participants, we switched the preparatory conditions to counterbalance the story sets. We illuminated the paper book with a light source, while the tablet had a backlight. Both the reflected light from the paper book and the incident light from the tablet were adjusted to 11.7 exposure value (EV at ISO = 400; equivalent to 2,100 lx) using an exposure meter (Flashmate L-308B; Sekonic Co. Ltd., Tokyo). The optical conditions of the surrounding environment were also controlled. The average time per page for self-paced reading was marginally longer under the Paper condition than the Tablet condition (Paper: 8.1 ± 2.4 s; Tablet: 7.5 ± 2.1 s; paired *t*-test: *t*[24] = 1.9, *p* = 0.07). Reading on a tablet involved instantaneous screen transitions with each page advance by minimally tapping the screen. To flip each page of the paper book, it took 0.7 ± 0.1 s (measured with three participants), which explains the time difference between the two conditions.

We prepared 6 questions each for stories 1, 2, 3, and 4 (Set 1 questions: 3, 4, 3, and 3, respectively; Set 2 questions: 3, 2, 3, and 3, respectively). For each question, there were four answer choices, in which we typically rated 2 points for the best choice, 1 point for the “second-best,” and 0 for the remaining choices (for 2 out of 24 questions, we set two second-best choices each).

For visual stimuli inside the scanner, an eyeglass-like MRI-compatible display (VisuaStim Digital; Resonance Technology Inc., Northridge, CA; resolution = 800 × 600, framerate = 60 fps) was used, and the lens powers were adjusted for nearsighted participants. The stimulus presentation was controlled by the Presentation software package (Neurobehavioral Systems, Berkeley, CA), which also collected behavioral data (accuracy rates and response times [RTs]). The participants provided their answers by pressing buttons on a response pad (HH-1x4-L; Current Designs Inc., Philadelphia, PA).

### Tasks

Under the Manga condition, each scanning run started with the presentation of a small red cross for eye fixation (5 s), followed by manga pages (20 s fixed for each double-page). A red cross was presented for 1 s after each page and for 5 s after the last page. During each presentation, participants rated their degree of empathy toward the protagonist depicted on the current manga page using a 4-point scale by pressing a button: 1) not empathetic at all, 2) somewhat empathetic, 3) empathetic, and 4) strongly empathetic. Participants were instructed to use the full range of ratings throughout the experiment.

Under the Questions condition, each scanning run started with the presentation of a red cross (5 s). A question and four answer choices (15 s) were visually presented, followed by a red cross (5 s); this sequence was repeated six times. During each presentation, participants chose the best answer by pressing the corresponding button. Separate runs were consecutively conducted in the following order (see Fig 1): i) manga reading for the second half (see Table 1) of story 1, ii) manga reading for the second half of story 2, iii) questions and answers for story 1, and iv) questions and answers for story 2. After a 10-minute break, four more runs were conducted for stories 3 and 4, following the same procedure inside the scanner. The participants received no explanation about the differences between Set 1 and 2 questions. Because the same happenings were repeatedly described in the manga (see the Introduction), differences in efforts during memory retrieval were minimized between Set 1 and 2 questions.

### MRI data acquisition and analyses

This study followed procedures that our team published previously [15–17]. For MRI data acquisition, the participant lay in a supine position with the head immobilized inside the radio-frequency coil. Imaging was performed using a 3.0T GE Signa HDxt system (GE Healthcare, Milwaukee, WI). Thirty axial slices were acquired using a gradient-echo echo-planar imaging (EPI) sequence [repetition time (TR) = 2 s, echo time (TE) = 30 ms, flip angle (FA) = 78°, field of view (FOV) = 192 × 192 mm², resolution = 3 × 3 mm²], with 3 mm thickness and a 0.5 mm interslice gap, covering the volume range of −38.5 to +66 mm along the anterior‒posterior commissure (AC‒PC) line. Each run comprised 103–124 volumes under the Manga condition and 67 under the Questions condition, both following four dummy scans to allow signal stabilization. After completion of the fMRI session, high-resolution T1-weighted images of the whole brain (136 axial slices, 1.0 × 1.0 × 1.0 mm³) were acquired using a three-dimensional fast spoiled gradient-recalled acquisition in the steady state (3D FSPGR) sequence (TR = 8.5 ms, TE = 2.6 ms, FA = 25°, FOV = 256 × 256 mm²). These structural images were used to normalize the functional data.

The fMRI data were analyzed in a standard manner using SPM12 statistical parametric mapping software (Wellcome Trust Centre for Neuroimaging, http://www.fil.ion.ucl.ac.uk/spm/) [18] implemented in MATLAB (Math Works, Natick, MA). Slice timing was corrected using the middle slice (the 15th slice chronologically) as the temporal reference. Time-series data from multiple runs were realigned to the first volume across all runs. The realigned data were resliced with 3 mm spacing using seventh-degree B-spline interpolation so that all voxels matched those of the first volume. A total of six runs from 5 participants were excluded due to motion exceeding 2 mm in translation or 1.4° in rotation along any axis.

After aligning to the AC‒PC line, each participant’s T1-weighted structural image was coregistered to the mean functional image generated during realignment. The coregistered structural image was then spatially normalized to the standard Montreal Neurological Institute (MNI) space using the “unified segmentation” algorithm with light regularization, which integrates tissue segmentation, bias correction, and spatial normalization into a single generative model [19]. The resulting deformation field was applied to the realigned functional data. Normalized functional images were smoothed using a 9 mm full-width at half-maximum isotropic Gaussian kernel. Low-frequency noise was removed by a high-pass filter with a 1/128 Hz cutoff.

In a first-level (fixed-effects) analysis, manga reading in each run under the Manga condition was treated as a single event (see Fig 1), and hemodynamic responses were modeled with a boxcar function of 188‒230 s (including the red cross presentation after each double-page) in duration according to each story. For each run under the Questions condition, hemodynamic responses were modeled using separate boxcar functions in Set 1 and Set 2 questions, each with a duration of 15 s. As a *control event* with no visual stimuli, the 5 s before and after each Manga or Questions event were separately modeled. The boxcar function was then convolved with a hemodynamic response function. To minimize the effects of head motion, the six realignment parameters obtained from preprocessing were included as a nuisance factor in a general linear model.

In the second-level (random-effects) analysis for intersubject comparisons, we used a flexible factorial design option (described below) with the following events: Manga, Questions (Set 1), and Questions (Set 2), as well as the corresponding control events (modeled separately for Manga and Questions). These five events were further divided according to the preparatory condition (Paper or Tablet). Additional nuisance covariates included age, LQ, and gender. We used whole-brain analysis to examine activated regions in an unbiased manner.

A repeated-measures analysis of variance (rANOVA) with post hoc *t*-tests was performed using two factors (preparatory condition and event), with a voxel-wise threshold of uncorrected *p* < 0.001 and a cluster-level threshold of *p* < 0.05 corrected using the false discovery rate (FDR). For supplementary analyses, we used family-wise error (FWE) correction for the cluster level. Consistent with standard use of the flexible factorial design (https://www.researchgate.net/publication/267779738), we assumed an equal variance among participants, as well as for both factors of preparatory condition and event. In all second-level contrasts, we subtracted out activations during individual control events in the Manga and Question runs; for simplicity, these subtractions are not explicitly mentioned in the main text. Exclusive masking was applied to remove negative activations in each contrast, using a voxel-level threshold of uncorrected *p* < 0.05. Anatomical identification of activated regions was performed primarily using the Anatomical Automatic Labeling (AAL) atlas (http://www.gin.cnrs.fr/en/tools/aal/) [20].

In addition to the whole-brain analyses, region-of-interest (ROI) analyses were conducted using the MarsBaR toolbox (http://marsbar.sourceforge.net/). Following standard methodology (https://marsbar-toolbox.github.io/marsbar.pdf), we selected ROIs as voxels within the clusters showing significant activation in the following contrasts: [Questions (Set 1 + Set 2) − Manga] and [Questions, Set 2 − Set 1], both for the Paper condition. Independent from these contrasts, we used those ROIs for the statistical comparisons shown in the Results section, such that there was no potential bias between signals for the Paper and Tablet conditions, as well as signals among the participants. For analyses of signal changes and behavioral data, we used R (https://www.r-project.org/).

To justify sample sizes in relation to what has been previously published, we performed a power analysis [21] by using our previous work with a multiple-choice task and a similar number of trials (16 trials) [6]. With an online estimator of Neuropower (http://www.neuropowertools.org/neuropower/neuropowerstart/), we used the whole-brain data from a group of 16 participants (Paper notebook group), and obtained average power of 99% for 25 subjects we tested (FDR corrected *p* < 0.05), which is above the typical aim of 80% power for fMRI.

## Results

### Behavioral data reflecting the Paper vs. Tablet preparatory conditions

**Fig 2:**
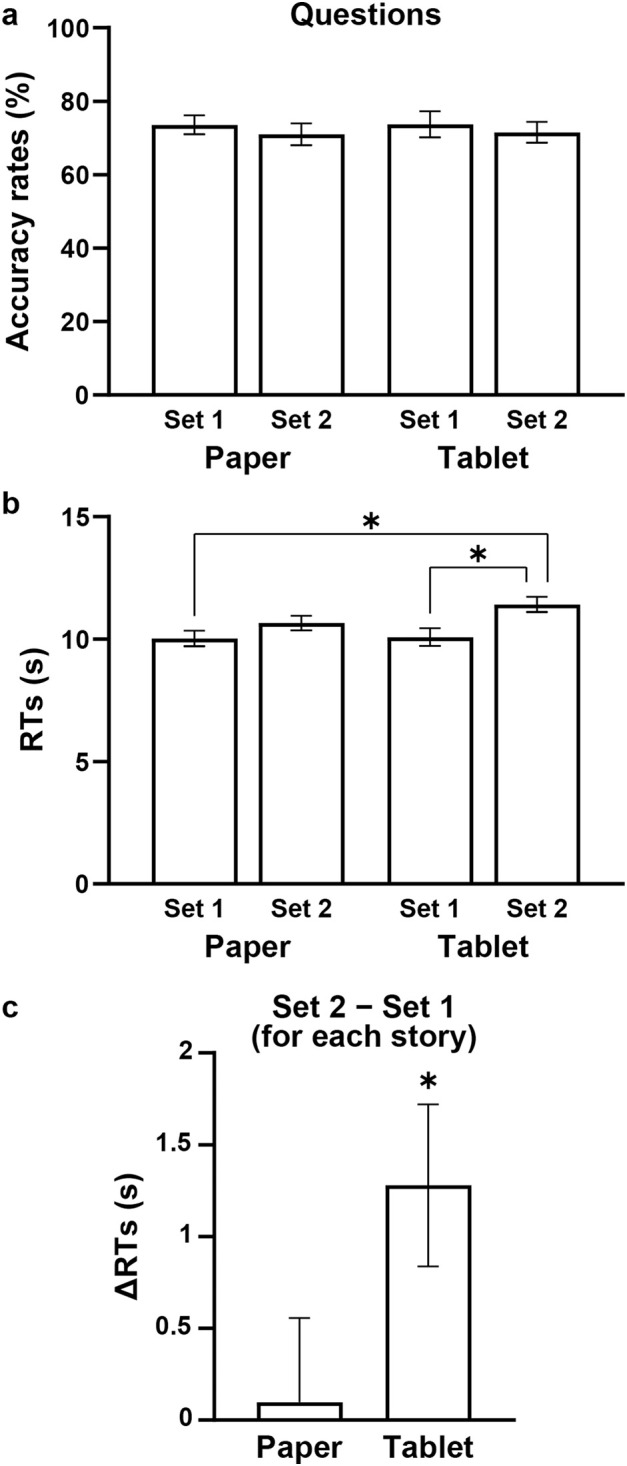
Behavioral data reflecting the Paper vs. Tablet preparatory conditions. **(a)** Accuracy rates for the questions, which were comparable between preparatory conditions (Paper and Tablet), as well as between Sets 1 and 2 (see the Introduction). **(b)** Response times (RTs) for the questions. Significantly longer RTs were observed in Set 2 for the Tablet condition. **(c)** Response time differences (ΔRTs) between questions Sets 1 and 2 (Set 2 − Set 1) for each story (see Table 1). Regarding correct answers, significant differences from zero were observed for the Tablet condition alone. Error bars indicate the SEM (standard error of the mean). *p < 0.05.

To examine differences in responses under the Questions condition between the Paper and Tablet preparatory conditions, as well as between Set 1 and Set 2 questions, we performed a two-way rANOVA with factors of preparatory condition [Paper, Tablet] and question set [Set 1, Set 2], comparing the accuracy rates and RTs (Fig 2). For accuracy rates, there were no significant effects (main effect of preparatory condition: *F*[1, 24] = 0.01, *p* = 0.9; main effect of question set: *F*[1, 24] = 0.7, *p* = 0.4; interaction of the main effects: *F*[1, 24] = 0.004, *p* = 0.9) (Fig 2a). In contrast, a two-way rANOVA for RTs showed a significant main effect of question set (*F*[1, 24] = 28, *p* < 0.0001), i.e., longer for *Set 2* (Fig 2b), as well as a marginal main effect of condition (*F*[1, 24] = 3.7, *p* = 0.07), while there was no significant interaction (*F*[1, 24] = 1.6, *p* = 0.2). With regard to multiple comparisons among the RTs, Tukey’s honestly significant difference (HSD) test confirmed that the RTs in Set 2 for the Tablet condition were significantly longer than in Set 1 for the Tablet condition (*q*[24] = 4.9, *p* = 0.01), as well as than in Set 1 for the Paper condition (*q*[24] = 5.1, *p* = 0.007), suggesting involvement of efficiency-based, rather than performance-based, elements.

To directly examine integration processes under the Questions condition, we compared the results of Sets 1 and 2 for *each story* (see Table 1). We focused on trials with correct answers and calculated response time differences (ΔRTs) between questions Sets 1 and 2 (i.e., Set 2 − Set 1) for each story (Fig 2c). While there was no significant difference from zero for the Paper condition (one-sample *t*-test, *t*(24) = 0.2, *p* = 0.8; Bonferroni corrected *α* = 0.025), we observed significant differences for the Tablet condition (*t*(24) = 2.9, *p* = 0.008). These results indicate *excessive* demands of linguistic and narrative-structural integration for correctly answering Set 2, when the first half was read under the Tablet condition.

To assess participants’ empathy while reading the *second* halves of the manga stories under the Manga condition, we divided each story into five sequential segments corresponding to 20%, 40%, 60%, 80%, and 100% of the narrative. A two-way rANOVA (preparatory condition [Paper, Tablet] and story progression [five levels]) revealed a significant main effect of story progression (*F*[1, 24] = 13, *p* < 0.0001) and a marginal main effect of preparatory condition (*F*[1, 24] = 3.7, *p* = 0.07; lower for the Paper condition), with no significant interaction (*F*[1, 24] = 0.1, *p* = 1). Under both conditions, the average empathy ratings at 80% and 100% were significantly higher than those at 20% and 40% (Paper: *t*[24] = 6.4, *p* < 0.0001; Table*t*: *t*[24] = 5.1, *p* < 0.0001), indicating active manga reading and appropriate comprehension by the participants.

### Differences in cortical activation patterns between Questions and Manga conditions

**Fig 3:**
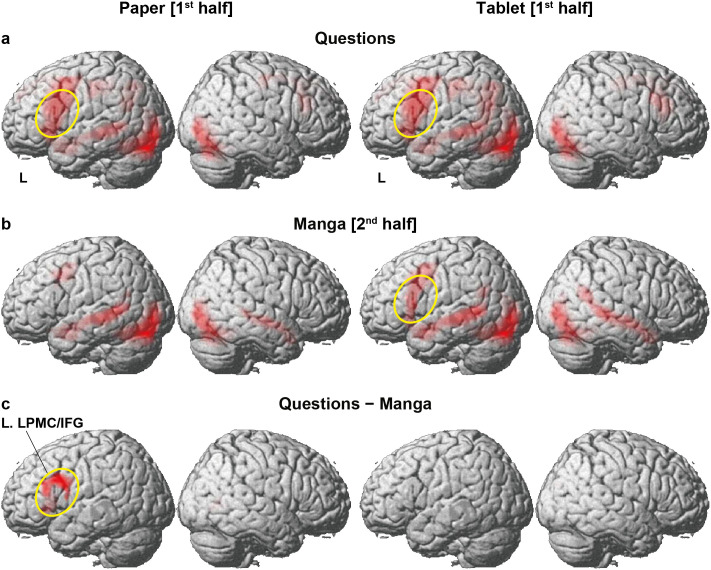
Differences in cortical activation patterns between Questions and Manga conditions. **(a)** Activations under the Questions condition (Sets 1 and 2 combined), separately for the Paper and Tablet preparatory conditions. **(b)** Activations under the Manga condition. **(c)** Activations selective to the Questions condition, compared with those under the Manga condition. Significant activation increases were observed in the left lateral premotor cortex and inferior frontal gyrus (L. LPMC/IFG) alone for the Paper condition. Activations in the same region of L. LPMC/IFG are highlighted by yellow ellipses in this and related panels. Significance was determined at uncorrected *p* < 0.001 for the voxel level and at false discovery rate (FDR)-corrected *p* < 0.05 for the cluster level.

Given the behavioral differences between the preparatory conditions, we next examined the cortical activation patterns associated with the Questions and Manga conditions. For the Questions collapsed across Sets 1 and 2, activation patterns were almost the same between the Paper and Tablet conditions (Fig 3a). The most notable activations appeared in the L. LPMC/IFG, R. LPMC/IFG, left superior/middle temporal gyri (L. STG/MTG), and L. AG. We also observed left-dominant bilateral occipito-temporal activations in the lingual/fusiform gyri (LG/FG), middle/inferior occipital gyri (MOG/IOG), and calcarine fissure, as well as cerebellar activations in the bilateral lobule IV/V/VI/Crus I and vermis IV/V/VI/VII. Additional activations were in the superior frontal gyrus, pre-supplementary motor area, and precuneus, as well as in the left hippocampus, basal ganglia, and thalamus.

In contrast, the Manga condition elicited activations basically similar to those under the Questions condition for the Tablet condition (Fig 3b), but activations in the L. LPMC/IFG became weaker. Moreover, the activation in the R. LPMC/IFG was below the threshold during the manga reading. The precuneus and bilateral hippocampi activations were observed for the Tablet condition alone, where medial-frontal and subcortical activations were absent for both conditions. Additional activations were observed in the R. STG/MTG extending along the superior temporal sulcus. Furthermore, for the Paper condition, frontal activations in Manga were localized in the L. LPMC alone.

Given the difference of activation patterns between the Questions and Manga conditions, we conducted a direct and highly stringent contrast [Questions – Manga]. Note that the Questions condition explicitly required greater processing demands of linguistic and narrative-structural integration, compared with the Manga condition that involved implicit reading alone. The Manga condition further controlled general cognitive loads: task engagement for continuous empathy ratings (see above), decision uncertainty, short-term memory to track the story line, memory retrieval, visual attention, and visual comfort for digital images. Significant activation emerged exclusively in the L. LPMC/IFG for the Paper condition (Fig 3c and Table 2), reflecting the relatively *lower* level of left frontal activations in Manga (note the absence of a yellow ellipse in Fig 3b, Paper condition). This result indicates that core processes for integration on the left frontal regions (see the Introduction) were reduced for the Paper condition while reading the *second* halves of manga stories. The reduced activations reflected less processing demands of integration for *both* halves, due to better interpretation of the *first* halves.

**Table 2:** Regions with activations selective to the Questions condition for each preparatory condition.

Brain region	BA	Side	Paper					Tablet				
			*x*	*y*	*z*	*Z*	Voxel	*x*	*y*	*z*	*Z*	Voxel
**Questions (Set 1 + Set 2) − Manga**												
LPMC/IFG	6/44	L	−54	14	32	4.0	303					
			−54	2	14	3.4	*					
IFG	44/45	L	−45	26	20	3.7	*					
	45/47	L	−27	26	−1	4.1	*					
**Questions, Set 2 − Set 1**												
LPMC	6/8/9	R	27	17	50	4.7	375					
IFG	44/45	R	42	14	26	4.4	*					
	45	R	45	35	23	3.9	*					
AG	39	R						39	−67	35	4.0	188

**Fig 4:**
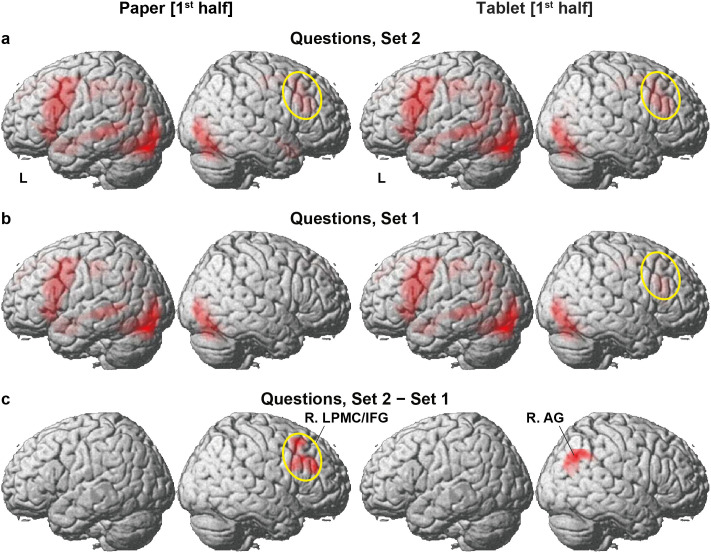
Differences in cortical activation patterns between question sets. **(a)** Activations during the presentation of Set 2 questions. Note the left-dominant activations in the language-related regions. **(b)** Activations during Set 1 questions. Activations in the language-related regions were clearly left-lateralized for the Paper condition. **(c)** Activations selective to Set 2, compared with Set 1. Significant activation increases were observed in the right lateral premotor cortex and inferior frontal gyrus (R. LPMC/IFG) for the Paper condition. Activations in the same region of R. LPMC/IFG are highlighted by yellow ellipses in this and related panels. Moreover, activations were observed in the right angular gyrus (R. AG) alone for the Tablet condition. Significance was determined at uncorrected *p* < 0.001 for the voxel level and at FDR-corrected *p* < 0.05 for the cluster level.

Stereotactic coordinates (*x*, *y*, *z*) in the MNI space are shown for activation peaks with *Z* values that were more than 16 mm apart (see Figs 3c and 4c). A region marked with an asterisk belongs to the same cluster as the region in the row directly above. BA: Brodmann’s area; L: left; R: right; AG: angular gyrus; IFG: inferior frontal gyrus; LPMC: lateral premotor cortex.

Regarding the reverse contrast [Manga − Questions], we observed activations in the R. STG/MTG for both Paper and Tablet conditions (S1 Fig), while those activations were relatively weaker for the Paper condition. This reduction is parallel to weaker activations of the L. LPMC/IFG in Manga for the Paper condition (see Fig 3b). Additional activations were observed in the right hippocampus and FG for the Tablet condition alone, suggesting increased general cognitive loads including memory retrieval during manga reading.

### Differences in cortical activation patterns between question sets

Given the significant behavioral differences between question sets, we next examined cortical activation patterns under the Questions condition as a function of question set and preparatory condition. In Set 2, where longer RTs were observed, lateral activations were left-dominant in the language areas of the L. LPMC/IFG and the L. STG/MTG/AG, whereas right activations were localized in the R. LPMC/IFG for both the Paper and Tablet conditions (Fig 4a). We also observed bilateral occipital and cerebellar activations, as shown in Fig 3a. For the Tablet condition, Set 1 elicited activations basically similar to those in Set 2, but activations became weaker in the R. LPMC/IFG (Fig 4b). For the Paper condition, in contrast, frontal activations were clearly left-lateralized.

Given the difference of activation patterns between Set 2 and Set 1, we conducted a direct contrast [Set 2 − Set 1]. This contrast would reveal the processing demands of integration for *both* halves of each manga story to answer Set 2 questions, strictly controlling efforts during memory retrieval (see the Tasks). Significant activation emerged exclusively in the R. LPMC/IFG for the Paper condition (Fig 4c and Table 2), reflecting the relatively *lower* level of right frontal activations in Set 1 (note the absence of a yellow ellipse in Fig 4b, Paper condition). This result indicates that *supportive* processes for integration on the *right* frontal regions (see the Introduction) were reduced in Set 1 for the Paper condition. For the Tablet condition, in contrast, significant activations were observed in the R. AG alone, consistent with the behavioral results of the longer RTs in Set 2. For the Tablet condition, we exploratorily validated R. AG activations in Set 2 at a lower threshold (uncorrected *p* < 0.01 for the voxel level).

### The distinct roles of the left and right LPMC/IFG

**Fig 5:**
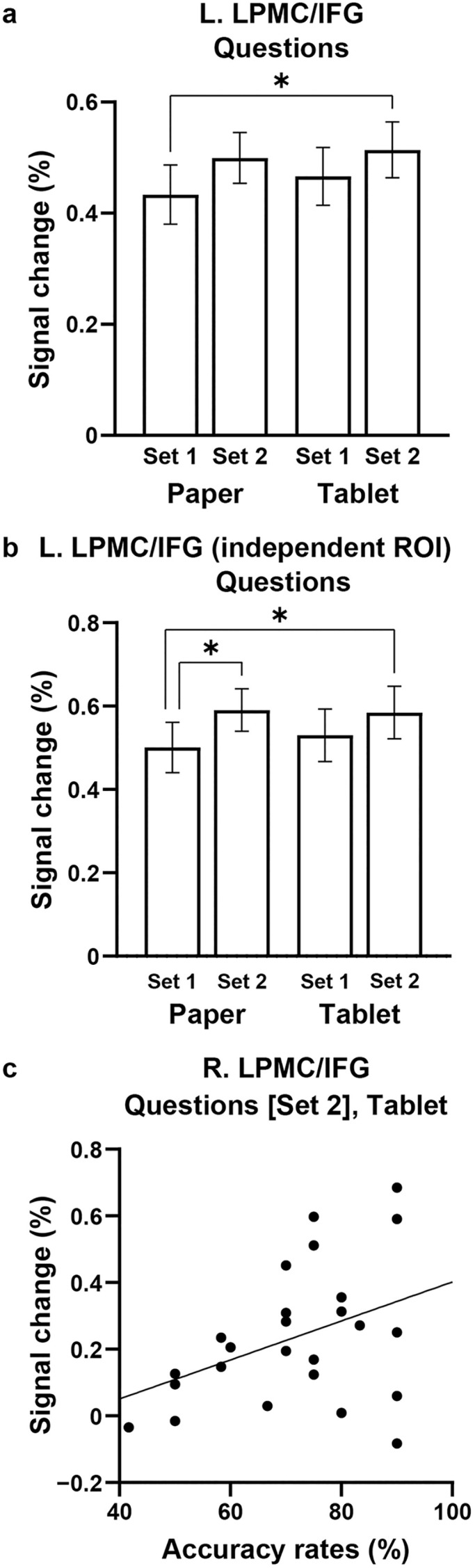
The distinct roles of the left and right LPMC/IFG. **(a)** Signal changes in the L. LPMC/IFG (taken from the yellow ellipse [Paper condition] in Fig 3c as a ROI) in each question set for each preparatory condition (Paper or Tablet). Activations in Set 2 for the Tablet condition were significantly higher than those in Set 1 for the Paper condition. **(b)** Replicated results for Fig 5a. An independent ROI for the L. LPMC/IFG was obtained from our previous fMRI study on language acquisition [Umejima K, Flynn S, Sakai KL. Sci Rep. 2024;14:54; Figure 4d of the cited paper]. Activations in Set 2 for both preparatory conditions were significantly higher than those in Set 1 for the Paper condition. **(c)** A significantly positive correlation between R. LPMC/IFG activations (taken from the yellow ellipse [Paper condition] in Fig 4c as a ROI) and accuracy rates under the Questions condition (Set 2 alone), observed for the Tablet condition (*r* = 0.40, *p* = 0.046). Error bars indicate the SEM. *p < 0.05.

We conducted ROI analyses on frontal cortex clusters that had been identified through direct comparisons between conditions and question sets. For the L. LPMC/IFG (see Fig 3c, Paper condition), a two-way rANOVA (question set and preparatory condition) for signal changes showed a significant main effect of question set (*F*[1, 24] = 4.6, *p* = 0.046), without a main effect of preparatory condition (*F*[1, 24] = 0.4, *p* = 0.5) or interaction (*F*[1, 24] = 0.2, *p* = 0.7) (Fig 5a). With regard to multiple comparisons, Tukey’s HSD test confirmed that the signal changes in Set 2 for the Tablet condition were significantly higher than in Set 1 for the Paper condition (*q*[24] = 3.9, *p* = 0.049). We conducted ROI analyses with an independent ROI of L. LPMC/IFG obtained from our previous fMRI study on language acquisition [22], where we identified activations for a new target language beyond accumulated linguistic knowledge in second and third languages. The above result was replicated by a two-way rANOVA (Fig 5b), with a significant main effect of question set (*F*[1, 24] = 6.4, *p* = 0.02), but without a main effect of preparatory condition (*F*[1, 24] = 0.09, *p* = 0.8) or interaction (*F*[1, 24] = 0.7, *p* = 0.4). Activations in Set 2 for both preparatory conditions were significantly higher than those in Set 1 for the Paper condition (*p* < 0.05). These results were partially consistent with the behavioral results (see Fig 2b), further revealing excessive integration processes for the Tablet condition.

We next focused on the R. LPMC/IFG (see Fig 4c, Paper condition). In Set 2 questions for the Tablet condition, we found a significantly positive correlation between signal changes in the R. LPMC/IFG and accuracy rates (*r* = 0.40, *p* = 0.046) (Fig 5c), with larger individual differences among participants with higher accuracy rates. This result indicates that the R. LPMC/IFG activations reflect excessive integration processes that support improved performance.

## Discussion

In the present study, we identified four key findings. First, behavioral results showed prospective effects of reading manga stories on a paper book or an electronic tablet, such that the RTs were prolonged in Set 2 questions for the Tablet condition (Fig 2b). By comparing the results of Sets 1 and 2 with correct answers for each story, we found significant response time differences for the Tablet condition alone (Fig 2c). These results indicate excessive demands of linguistic and narrative-structural integration for correctly answering Set 2, when the first half was read under the Tablet condition. Secondly, for the Paper condition, activations in the L. LPMC/IFG significantly decreased under the Manga condition while reading the second halves for the memorized stories (Fig 3). Activations in the R. LPMC/IFG also decreased in Set 1 for the Paper condition (Fig 4). These reduced activations were observed by direct contrasts controlling general cognitive factors, indicating saved processing demands for the Paper condition. Thirdly, core activations in the L. LPMC/IFG were highest in Set 2 for the Tablet condition (Fig 5a and 5b), while supportive activations in the R. LPMC/IFG correlated with individual accuracy rates in Set 2 for the Tablet condition (Fig 5c), indicating that excessive integration processes support improved performances required by correct answers (see Fig 2c). The present results demonstrate stronger prospective effects of reading on paper books, such that linguistic and narrative-structural integration processes are facilitated and led to saved excessive activations.

The positive correlation between R. LPMC/IFG activations and performance for the Tablet condition parallels the L. IFG activations that predicted individual gains in second language acquisition during its early stage [23,24]. Regarding the functional connectivity between these bilateral regions, we previously identified three syntax-related networks in the brain [25], each centered on a distinct frontal language area. Network I involves the opercular and triangular parts of the L. IFG (BA 44/45). Network II centers on the L. LPMC (BA 6/8). Network III is anchored in the triangular and orbital parts of the L. IFG (BA 45/47). These three networks represent anatomically and functionally distinct modules related to syntactic processing. Our present findings suggest that both the L. IFG and R. LPMC/IFG, which are components of Network I, are differentially recruited depending on preparatory reading quality and linguistic integration processes.

A previous study using a verb generation task identified activations in the L. IFG when generating normal (i.e., semantically proximate) predicate-argument relations, whereas activations were observed in the right middle frontal gyrus (BA 8/9) when generating relatively unusual (i.e., semantically distant) relations [26]. It has been hypothesized that the left and right regions are related to focused and diffuse semantic fields, respectively [27]. However, we can indicate an alternative account, such that the left region became involved in the *propositional* system of making a basic theta-structure [NB: theta-structure means structure of a predicate and its arguments with semantic roles] [11], and that the right region was recruited for supportive linguistic processes. Beyond these frontal regions, our data further highlight the contribution of the right parietal and temporal areas involved in manga reading.

The enhanced activations in the R. AG for Set 2 (Fig 4c, Tablet condition) may reflect the increased load of reconstructing the spatial layout of individual frames of both halves on a double-page spread. The dorsal portion of the R. AG activations overlapped with the R. AG/intraparietal sulcus (IPS) region identified in a comparison between double- and single-page manga presentations [7], corresponding to the region associated with hemispatial neglect. Another possibility is that the dorsal R. AG is a portion of the R. IPS/AG within the syntax-related Network I, which shows activation under higher syntactic load [28]. Further studies are needed to clarify the functional specialization of these right parietal regions.

Activations in the R. STG/MTG were observed under the Manga condition but not under the Questions condition (Fig 3ab and S1 Fig). One possible role of this region is spatial processing during manga reading. In a study using repetitive transcranial magnetic stimulation in healthy participants, disruption of the R. STG caused increased errors in localizing a vertical line within a cross-shaped visual stimulus [29]. Another possibility is that this region supports the tracking of frame sequences during manga reading, as shown by activations in the bilateral STG/MTG and dorsal portion of the cerebellum when four-frame manga presentations were compared between sequentially coherent and randomized orders [30]. More specifically, activations in the R. STG/MTG and dorsal cerebellum may reflect scene construction and spatial sequencing, involving spatial mapping of narratives, visual coherence processing, and reordering/reconstructing visual narratives, all leading to schema-level processing. It is also likely that the R. STG/MTG activations reflect greater demands of semantic processing in Manga than those in Questions, where manga images contain richer information than individual words [10]. Among the regions commonly activated under the Manga and Questions conditions, a corresponding cerebellar region was consistently observed in our previous study [7], where manga reading on a double-page spread with preserved structures was contrasted with spreads composed of mixed and randomly ordered stories. The right dorsal cerebellum, overlapping this region, belongs to Network II [28], suggesting a role in representing information in correct temporal sequence.

As regards the occipito-temporal activations, studies on dyslexia have identified that the bilateral FG/LG and MOG/IOG are included in the responsible areas for written language processing [31]. The L. FG is particularly involved in associating orthography and phonology beyond mere association of sound and symbol in healthy participants [32]. A possible general role of the L. FG/LG/MOG would be to distinguish similar elements of the same category, which is crucial for making the linear order of grouped noun phrases or verbs in a sentence [33]. In the present study, we observed left-dominant activations in the FG/LG and MOG/IOG in both Questions and Manga (see Figs 3 and 4), where the right homologs may have helped precise identification of the expressions in a visual narrative.

As noted in the Introduction, the advantages of paper books include “spatiotemporally fixed cues to the length of the text” [2], which are also crucial in manga reading. This physical feature is particularly important for associating new structural information with stable double-page spreads as predictable spatial anchors on a paper book. Such information supports the consolidation of narrative content including its story schema, and allows facilitation of generative processes including later integration, which explains reduced RTs and saved frontal activations based on paper reading. It should be noted that the frame structure (i.e., the relative positions of frames) on each double-page spread is embedded within a broader spatiotemporal framework defined by the physical sequence of pages, constituting a recursive structure unique to manga. Moreover, paper books facilitate the perception of informational flow across frame structures and pages by preserving consistent visual and tactile cues, i.e., providing perceptual continuity, thereby enabling deeper immersion in the storyline and its interpretation, such as the inner thoughts and feelings of protagonists. Reading on a tablet, on the other hand, involves instantaneous screen transitions with a mechanical/automatic page-turner, which may disrupt a unified reading experience. Additionally, reading with paper books and tablet differs in reflected versus backlit viewing, which also possibly affect reading experience, as indicated by the comparison between “light-on” and “light-through” for the acceptance of a film [34]. In conclusion, the results demonstrate that the spatiotemporal and structural properties of manga enhance linguistic, narrative-structural, and schema-related interpretation, and that these properties are more effectively accessed in the brain when reading paper books than when using electronic devices.

## Supporting information

S1 Fig Activations selective to the Manga condition, compared with those under the Questions condition. Significant activation increases were observed in the right superior/middle temporal gyri (R. STG/MTG) for both Paper and Tablet conditions. Parasagittal sections at *x* = 33 in MNI coordinates showed additional activations in the right hippocampus and right fusiform gyrus (R. FG) for the Tablet condition alone. Significance was determined at uncorrected *p* < 0.001 for the voxel level and at family-wise error (FWE)-corrected *p* < 0.05 for the cluster level.
(TIF)
