# Supplementary figures and images for: Manga reading on paper vs. digital devices: Prospective effects on core and supportive integration processes in the brain
Source: PLoS One. 2026 Jun 3;21(6):e0349778. doi: 10.1371/journal.pone.0349778 (PMC13232794; doi:10.1371/journal.pone.0349778)

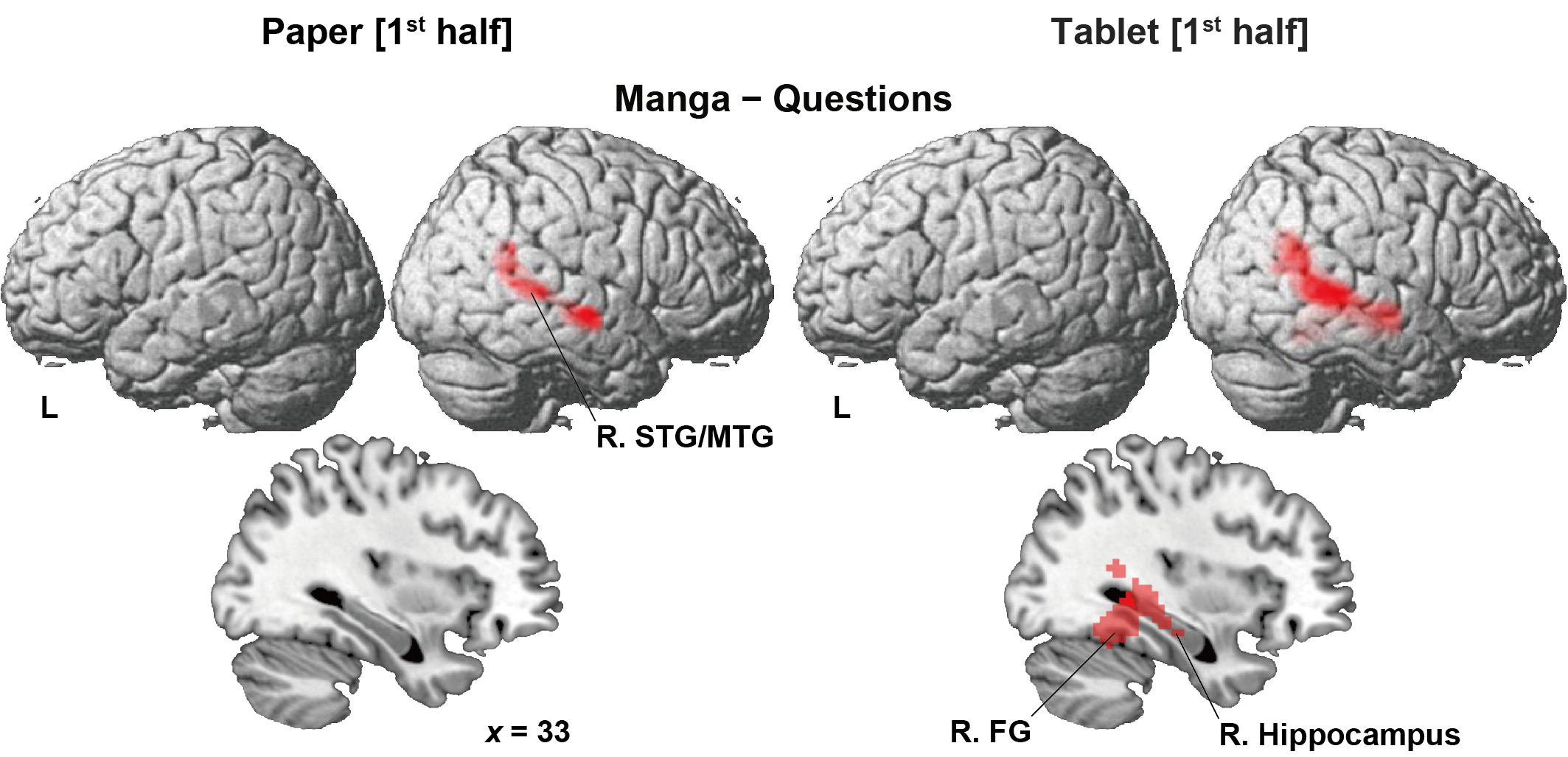

Supplement: S1 Fig — Significant activation increases were observed in the right superior/middle temporal gyri (R. STG/MTG) for both Paper and Tablet conditions. Parasagittal sections at x = 33 in MNI coordinates showed additional activations in the right hippocampus and right fusiform gyrus (R. FG) for the Tablet condition alone. Significance was determined at uncorrected p < 0.001 for the voxel level and at family-wise error (FWE)-corrected p < 0.05 for the cluster level. (TIF) [file pone.0349778.s001.tif]
